# α-Synuclein Toxicity in *Drosophila melanogaster* Is Enhanced by the Presence of Iron: Implications for Parkinson’s Disease

**DOI:** 10.3390/antiox12020261

**Published:** 2023-01-24

**Authors:** Francesco Agostini, Luigi Bubacco, Sasanka Chakrabarti, Marco Bisaglia

**Affiliations:** 1 Department of Biology, University of Padova, Via Ugo Bassi 58/B, 35131 Padova, Italy; 2 Study Center for Neurodegeneration (CESNE), 35121 Padova, Italy; 3 Department of Biochemistry and Central Research Laboratory, Maharishi Markandeshwar Institute of Medical Sciences and Research, Maharishi Markandeshwar University (Deemed to Be), Mullana, Ambala 133207, India

**Keywords:** *Drosophila melanogaster*, ferroptosis, iron, Parkinson’s disease, α-synuclein

## Abstract

Parkinson’s disease (PD) is the second most common neurodegenerative disorder, characterized by the preferential loss of dopaminergic neurons and by the accumulation of intracellular inclusions mainly composed of α-synuclein (α-Syn). While the etiopathogenesis of the disorder is still elusive, recent experimental evidence supports the involvement of ferroptosis, an iron-dependent cell death pathway, in the pathogenesis of PD. In the present work, using different ferroptosis inducers and inhibitors, we evaluated, in vivo, the involvement of iron in the α-Syn-mediated toxicity. Using a *Drosophila melanogaster* model of PD based on the selective over-expression of α-Syn within dopaminergic neurons, we demonstrated that the over-expression of α-Syn promotes the accumulation of protein aggregates, which is accompanied by dopaminergic neurodegeneration, locomotor impairment, and lifespan reduction. These pathological phenotypes were further exacerbated by reduced intracellular levels of glutathione or increased concentrations of iron. Coherently, both the use of an iron chelator and the presence of the antioxidant compound N-acetylcysteine exerted protective effects. Overall, our results support the involvement of ferroptosis in the α-Syn-mediated toxicity.

## 1. Introduction

Parkinson’s disease (PD) is a progressive age-related neurodegenerative disorder, clinically defined by the presence of motor symptoms, including muscular rigidity, resting tremor, postural instability, and bradykinesia. One of the pathological hallmarks of PD is the preferential degeneration of dopaminergic neurons in the substantia nigra pars compacta, which is responsible for the clinical motor features of the disorder. PD is mostly a sporadic disease, but around 5–10% of all cases have a genetic origin [1]. Among the proteins associated with the familial forms of the disorder, α-synuclein (α-Syn) plays a prominent role. Not only has the protein been the first to be linked to genetic forms of PD [2], but, most importantly, it is also one of the main components of the cytosolic inclusions referred to as Lewy bodies and Lewy neurites, which represent another pathological hallmark of the disorder [3].

PD is still an incurable disorder, and the main therapeutic approach is based on L-DOPA treatment, a pharmacological therapy introduced 60 years ago [4] that provides only symptomatic relief without hampering the progression of the disease. One of the major concerns in the definition of novel therapies is the lack of a complete understanding of the pathological processes involved in the etiopathogenesis and progression of PD. Although the pathogenesis of PD is still elusive, a general agreement exists on the involvement of oxidative injury, mitochondrial dysfunction, neuroinflammation, and proteostasis impairment in the progression of the disease. Recently, a new non-apoptotic regulated form of cell death, referred to as ferroptosis, has been described [5] that could rationalize the involvement in the disorder of all the processes mentioned above. Ferroptosis is an iron-dependent cell death pathway that involves the accumulation of lipid peroxidation, accompanied by the concomitant depletion of intracellular reduced glutathione (GSH). Of note, several pathological phenotypes related to PD also constitute key features of ferroptosis [6,7]. For instance, iron accumulation is enhanced in the substantia nigra of PD patients and appears to correlate with disease severity [6,8,9], while a concomitant specific decrease in GSH levels has been reported in the same brain region of PD patients [10,11]. Moreover, additionally, elevated levels of lipid peroxidation were measured in the postmortem PD brain [12,13]. Altogether, these observations represent a strong indication of the involvement of ferroptosis in the neurodegenerative processes observed in PD.

The main cellular protective mechanism against ferroptosis depends on the enzyme glutathione peroxidase 4 (GPX4), which selectively transforms phospholipid hydroperoxides into lipid alcohols using the tri-peptide glutathione (GSH) as a cofactor [14]. Being the rate-limiting amino acid utilized in the synthesis of GSH, cysteine plays an essential role in cellular antioxidative defense [15]. Its cytosolic accumulation strongly depends on the cystine/glutamate antiporter, system X_c_^−^, which imports cystine across the cell membrane into the cytosol, where it is reduced to cysteine. Several substances have been described and used to modulate ferroptosis and their use has been fundamental to investigating ferroptosis-related mechanisms in different experimental models [7,16]. For instance, since the accumulation of lipid peroxides in ferroptosis is iron-dependent, it can be inhibited by iron chelators, such as deferiprone (DEF) and deferoxamine, small lipophilic antioxidants, including ferrostatin, liproxstatin, and vitamin E, and other antioxidant molecules affecting GSH intracellular levels, such as N-acetylcysteine (NAC). Conversely, in addition to increased cytosolic iron concentration, ferroptosis can be induced by altering the X_c_^−^/GSH/GPX4 axis. In this frame, erastin and RSL3 are often used to inhibit X_c_^−^ and GPX4, respectively.

In the present work, using both ferroptosis inducers and inhibitors on a *Drosophila melanogaster* model of PD based on the selective over-expression of α-Syn within dopaminergic neurons, we evaluated, in vivo, the involvement of iron in the α-Syn-mediated toxicity. Overall, in agreement with very recent results obtained in vitro in pathological relevant cellular models of α-synucleinopathies, our results support the recently proposed idea that ferroptosis participates in the pathogenesis of PD.

## 2. Materials and Methods

Drosophila strains and culture maintenance: Flies were raised on agar, cornmeal, and yeast food, at 25 °C in 12-h light/dark cycles. Only male flies were used in all experiments. The following strains were obtained from the Bloomington *Drosophila* Stock Center: *w^1118^* (#5905), UAS-*SNCA*/Cyo (#51375), TH-GAL4 (#8848).

*Lifespan*: Groups of 20 fly males were collected under brief CO_2_ exposure and placed in fresh food vials containing the ferroptosis modulators at the final desired concentrations. Flies were transferred to fresh food vials containing ferroptosis modulators every 3–4 days, and the number of dead flies was counted daily. The percentage of survival was calculated at the end of the experiments.

*Locomotion assay*: Groups of 20 fly males were collected under brief CO_2_ exposure and placed in fresh food vials, and the locomotion behavior was assessed after 7, 14, and 21 days. During this period, food was changed every 2–3 days. The mobility of flies from each treatment group was assessed through a negative geotaxis climbing assay using a counter-current apparatus with six tubes in the lower frame and five in the upper frame. Flies were placed in the first plastic vial (1.5 cm diameter and 10 cm height) and gently tapped to the bottom. After 10 s, the upper frame was moved to the right, and the flies that passed in the upper tubes during this period were transferred to the next lower tubes by gently tapping. This procedure was repeated five times. For each genotype, the climbing index was calculated using the following formula:

CI = [(#F5 × 5) + (#F4 × 4) + (#F3 × 3) + (#F2 × 2) + (#F1 × 1) + (#F0 × 0)/(#FT)]

where #Fn is the number of flies in the tube, “n” (being 0 in the initial tube and 5 in the last tube), and #FT is the total number of flies.

*Immunofluorescence and imaging*: One and 3-week-old adult fly brains were labeled with anti-Tyrosine Hydroxylase (TH) (rabbit, EMD Millipore, AB152, Temecula, MA, USA) and anti-aggregated α-Syn (mouse, Sigma Aldrich, Burlington, MA, USA, clone 5G4, MABN389). In brief, after dissection, brains were fixed in 0.4% paraformaldehyde (PFA) for 1 h at room temperature (RT). After permeabilization in 1% triton dissolved in PBS for 10 min at RT and blocking in 1% BSA, 0.3% triton dissolved in PBS for 1 h at RT, brains were incubated overnight with primary antibodies diluted in PBS plus 0.3% triton, and 0.1% BSA at 4 °C. Then, brains were incubated with the fluorophore-conjugated secondary antibodies for 2 h at RT. Finally, brains were mounted in Mowiol^®^ 4–88 (Calbiochem, San Diego, CA, USA, 475904). The number of posterior dopaminergic neurons was counted manually in each brain hemisphere using a Leica DMI4000B inverted fluorescence microscope (Leica Microsystems, Heerbrugg, Switzerland). Importantly, to control for investigator bias, all experiments were carried out with the experimenter blinded to the sample genotypes or treatment throughout the analysis. To detect α-Syn-positive signals, images were acquired using a LeicaSP5 confocal microscope (Leica Microsystems), and α-Syn-positive dopaminergic neurons were counted in each brain hemisphere.

*Statistical analysis*: Data were collected from at least three independent experiments. Graphs were produced and statistical analyses were performed using GraphPad Prism 9 software. The statistical difference among mean values was assessed by the Mann–Whitney U test or one-way ANOVA, followed by Tukey’s multiple comparisons post hoc test, for grouped comparisons. Survival analysis was performed by Mantel-Cox log-rank test. *p* values < 0.05 were considered significant.

## 3. Results

### 3.1. Fly Lifespan

**Figure 1:**
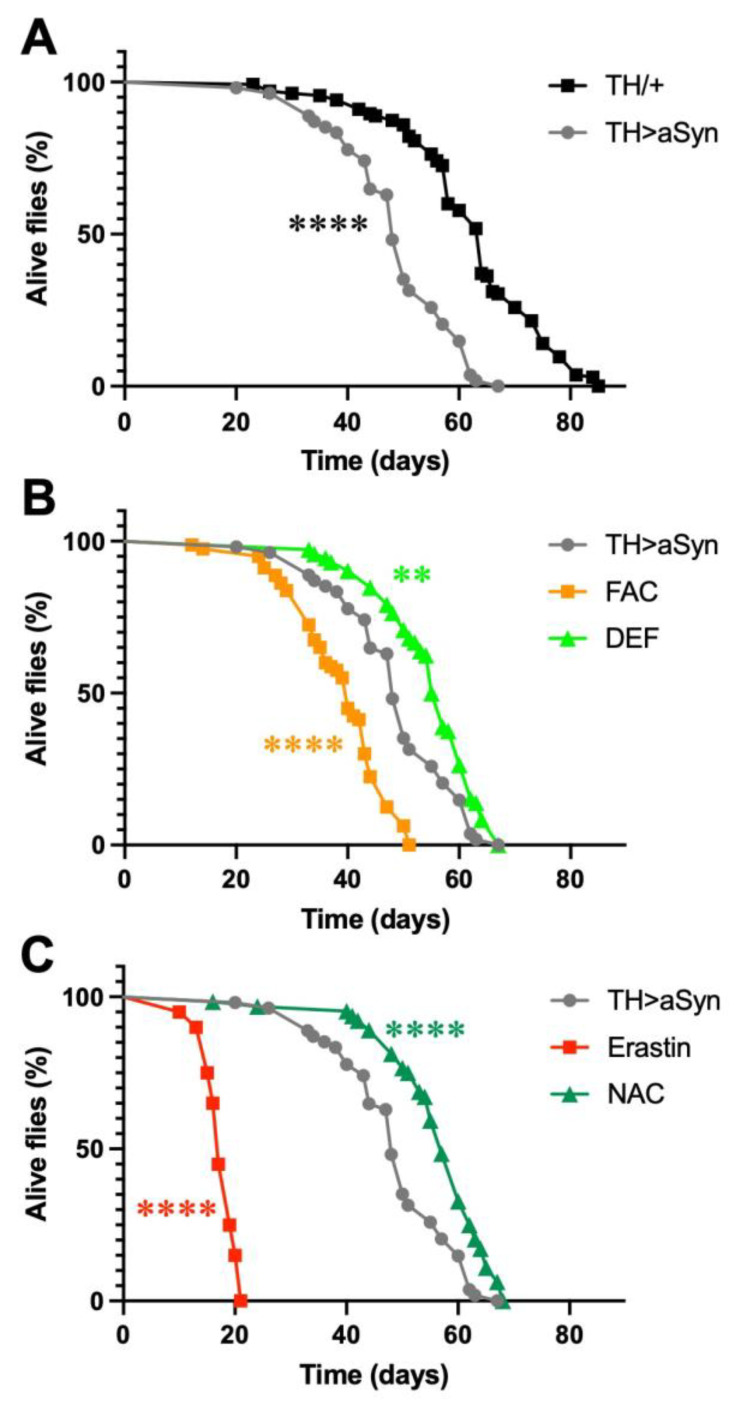
Ferroptosis modulators affect α-Syn-induced toxicity. (**A**) Lifespan analysis. α-Syn mutants lived significantly less than control flies. (**B**) Lifespan analysis. FAC significantly enhanced α-Syn-induced toxicity while DEF significantly protected flies against α-Syn-dependent toxicity. (**C**) Lifespan analysis. Erastin significantly enhanced α-Syn-induced toxicity, while NAC significantly protected flies against α-Syn-dependent toxicity (Mantel–Cox log-rank test: **** and ** indicate *p* < 0.0001, and <0.01). N: 135 TH/+, 54 TH > α-Syn, 80 FAC, 72 DEF, 20 Erastin, and 64 NAC.

In this work, to selectively over-express α-Syn within dopaminergic neurons, we exploited the GAL4/UAS system, which consists of two parts: the yeast transcription activator protein GAL4 and the upstream activating sequence (UAS), to which GAL4 specifically binds to activate transcription. Both are absent in flies. The GAL4 gene has been placed under the control of the *Tyrosine Hydroxylase* (*TH*) gene promoter, which drives the selective expression of GAL4 within dopaminergic neurons, while the UAS controls the expression of the *α-Syn* gene. In particular, we used a previously generated and characterized *Drosophila* strain that expresses insect codon-optimized high levels of human α-Syn [17]. Previous work with this line demonstrated that the selective over-expression of α-Syn in dopaminergic neurons was able to induce, in 20-day-old flies, a significant reduction in the number of TH-positive dopaminergic neurons in the protocerebral posterior lateral 1 (PPL1) cluster, relative to the control [17]. In the first series of experiments of this work, we measured the lifespan of this strain and compared it with control flies bearing the same genetic background. We observed that the selective over-expression of α-Syn in dopaminergic neurons strongly reduces the lifespan of flies with a shift of median survival (t_1/2_) from 64 to 48 days (Figure 1A). Then, to gain insights into the possible involvement of ferroptosis in α-Syn-mediated toxicity, we carried out experiments by over-expressing α-Syn in the presence of either ferroptosis-inducing molecules or anti-ferroptosis compounds. For each compound, the concentration utilized was chosen on the basis of previously published works [18,19,20,21]. First, we tested the effect of iron on α-Syn-dependent toxicity. To this aim, we treated flies over-expressing α-Syn with either 5 mM ferric ammonium citrate (FAC), which increases the endogenous levels of iron [18], or 1 mM DEF, an iron chelator. Interestingly, as represented in Figure 1B, we observed that an increased amount of iron further decreased the fly lifespan (t_1/2_ = 40 days), while a reduced concentration of this metal ion partially rescued the toxic effects of α-Syn (t_1/2_ = 56 days). We then evaluated the effects of two molecules able to modulate the levels of GSH by treating flies with either 10 μM erastin, an inhibitor of the cystine-glutamate transporter (system X_c_^−^), which inhibits the synthesis of GSH [22], or 0.1 mM NAC, a precursor for GSH biosynthesis under conditions of GSH deficiency [23]. As shown in Figure 1C, a strong decrease in the lifespan of α-Syn over-expressing flies was observed following the erastin treatment (t_1/2_ = 17 days), while the presence of NAC partially rescued the α-Syn-dependent toxicity (t_1/2_ = 57 days).

### 3.2. Fly Locomotion

**Figure 2:**
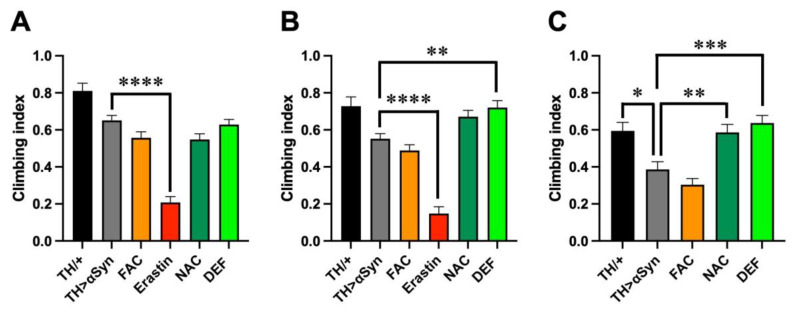
Ferroptosis modulators affect α-Syn-induced locomotor impairment. (**A**) Climbing activity (mean ± SEM) performed in 1-week-old flies. Analysis of variance and Tukey’s multiple comparisons post-hoc test indicate the following statistically significant differences: *** TH/+ vs. FAC, **** TH/+ vs. Erastin, *** TH/+ vs. NAC, * TH/+ vs. DEF, **** TH > αSyn vs. Erastin. N: 39 TH/+, 163 TH > α-Syn, 130 FAC, 53 Erastin, 139 NAC, and 145 DEF. (**B**) Climbing activity (mean ± SEM) performed in 2-week-old flies. Analysis of variance and Tukey’s multiple comparisons post-hoc test indicate the following statistically significant differences: ** TH/+ vs. FAC, **** TH/+ vs. Erastin, **** TH > αSyn vs. Erastin, ** TH > αSyn vs. DEF. N: 39 TH/+, 205 TH > α-Syn, 129 FAC, 42 Erastin, 93 NAC, and 78 DEF. (**C**) Climbing activity (mean ± SEM) performed in 3-week-old flies. Analysis of variance and Tukey’s multiple comparisons post-hoc test indicate the following statistically significant differences: * TH/+ vs. TH > αSyn, *** TH/+ vs. FAC, ** TH > αSyn vs. NAC, *** TH > αSyn vs. DEF. N: 37 TH/+, 74 TH > α-Syn, 89 FAC, 20 Erastin, 68 NAC, and 60 DEF. ****, ***, **, *, indicate *p* < 0.0001, <0.001, <0.01, <0.05. For the sake of clarity, only the statistically significant differences between untreated α-Syn over-expressing flies and the other conditions are reported in each graph.

As motor impairment is the main clinical hallmark of PD, we then compared the locomotor behavior through a negative geotaxis-based climbing assay. Considering the different median survival periods previously observed, to obtain a more precise description of the motor defects, we carried out the analysis at different time intervals, namely in 1-, 2-, and 3-week-old flies. Although at 1 week after the eclosion we did not observe fly death in any genotype considered and with any treatment applied, the over-expression of α-Syn in dopaminergic neurons slightly affected the climbing ability of flies with respect to controls, although the difference was not statistically significant (Figure 2A). Interestingly, every treatment used was unable to modulate the effects induced by α-Syn with the exception of erastin, in line with the high toxicity previously observed in the presence of this pro-ferroptosis molecule. A similar trend was also observed in the 2-week-old flies (Figure 2B). Additionally, in this case, the treatment with erastin strongly enhanced the toxicity derived from the presence of α-Syn. It is worth mentioning that since, at this time point, almost 20% of flies were already dead, the analysis was performed with, and refers to, survivors. In contrast to the observations performed 1 week after eclosion, in 2-week-old flies, the treatment with FAC also induced locomotor defects in comparison with control flies, although not at the level of erastin. Moreover, the presence of NAC or DEF exerted a protective role in the climbing activity, which was very similar to control flies. Slightly more pronounced differences emerged in 3-week-old flies (Figure 2C). At this time point, the climbing ability of our control was also slightly affected with respect to 1-week-old flies, but the effects were more evident in flies overexpressing α-Syn, with and without the addition of FAC in the food, while, once again, NAC and DEF exerted a protective action. It is worth mentioning that, in this time frame, the climbing assay was not performed in flies treated with erastin, because no survivors were present, while 3 weeks after the eclosion, less than 5% of dead flies were counted for the other treatments.

### 3.3. Dopaminergic Neuronal Degeneration

**Figure 3:**
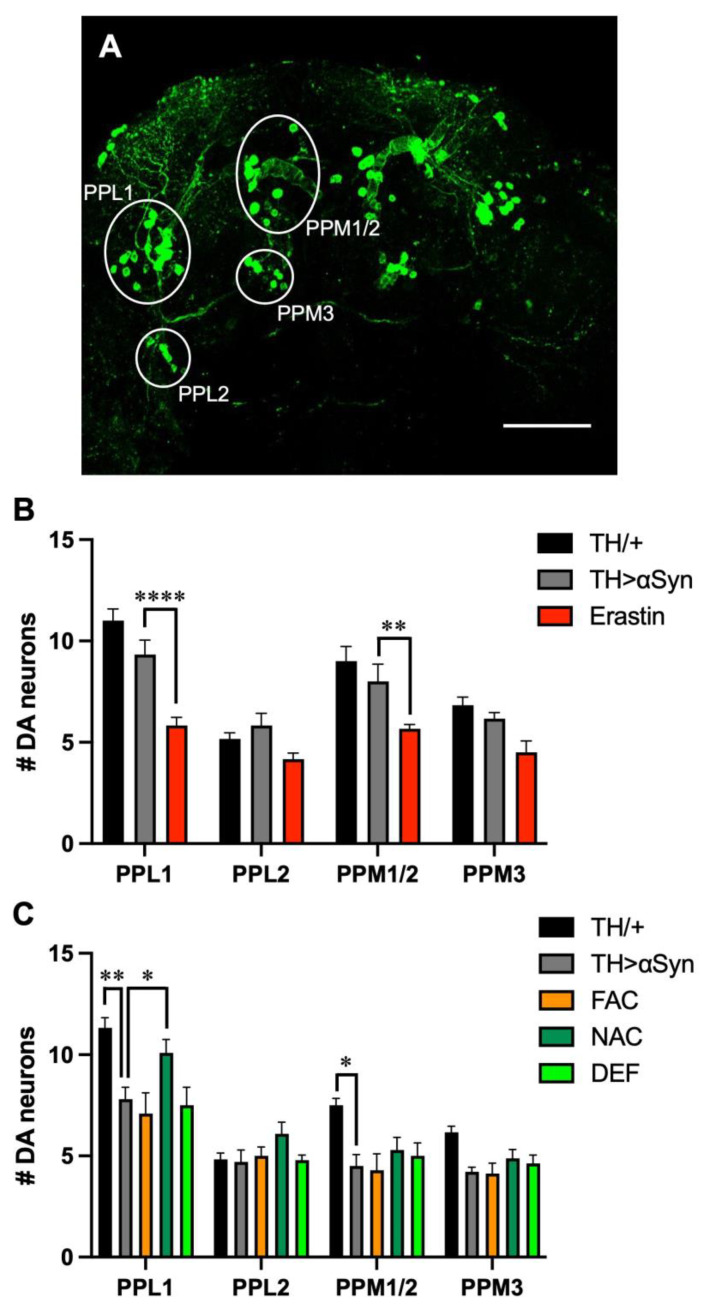
Influence of ferroptosis modulators on α-Syn-induced dopaminergic neurodegeneration. (**A**) Representative immunofluorescent image, acquired on 1-week-old control (TH/+) flies, showing the localization and name of the main clusters of protocerebrum posterior dopaminergic neurons. Scale bar 50 μm (**B**) Quantitative analysis (mean ± SEM) was performed in 1-week-old control, untreated αSyn-overexpressing flies and following the treatment with Erastin. Analysis of variance and Tukey’s multiple comparisons post-hoc test indicate the following statistically significant differences: (PPL1) **** TH/+ vs. Erastin, **** TH > αSyn vs. Erastin; (PPM1/2) *** TH/+ vs. Erastin, ** TH > αSyn vs. Erastin; (PPM3) ** TH/+ vs. Erastin. (**C**) Quantitative analysis (mean ± SEM) was performed in 3-week-old control, untreated αSyn-overexpressing flies and following the treatment with FAC, NAC, and DEF. Analysis of variance and Tukey’s multiple comparisons post-hoc test indicate the following statistically significant differences: (PPL1) ** TH/+ vs. TH > αSyn, *** TH/+ vs. FAC, *** TH/+ vs. DEF, * TH > αSyn vs. NAC; (PPM1/2) * TH/+ vs. TH > αSyn, ** TH/+ vs. FAC. ****, ***, **, *, indicate *p* < 0.0001, <0.001, <0.01, <0.05. For the sake of clarity, only the statistically significant differences between untreated α-Syn over-expressing flies and the other conditions are reported in each graph.

Since motor symptoms associated with PD are the consequence of the preferential degeneration of dopaminergic neurons, we then evaluated the loss of TH-positive neurons in our experimental models [24]. In the protocerebrum posterior of flies, dopaminergic neurons are grouped in clusters with bilateral symmetry (Figure 3A), and as usually done in *Drosophila* models of PD [24], we considered the following clusters: protocerebral posterior lateral 1 and 2 (PPL1 and PPL2), and protocerebral posterior medial 1/2 and 3 (PPM1/2 and PPM3). Considering that the treatment with erastin strongly affected the locomotor behavior of flies over-expressing α-Syn already 1 week after eclosion, in the presence of this ferroptosis-inducing molecule, the analysis was carried out in 1-week-old flies. Interestingly, the presence of erastin strongly promoted dopaminergic loss in most of the neuronal clusters considered. Moreover, although the effects were not statistically significant with any dopaminergic cluster considered, a neurodegenerative trend was observed also in the α-Syn expressing strain with respect to control flies (Figure 3B).

This result is in line with the first work, in which the investigators observed no loss of TH-positive neurons in 1-day-old flies, while a reduction in PPL1 cluster was present in 20-day-old individuals [17]. Since in α-Syn over-expressing flies, in the absence or in the presence of the other compounds considered in this work, the climbing defects appeared in 3-week-old flies, we then repeated the analysis 3 weeks after eclosion. With our experimental set-up, the intensity of the signals corresponding to dopaminergic neurons was lower in our aged control flies with respect to young individuals, even in the absence of neuronal loss (data not shown). It is possible that the loss of signal intensity precedes cell death. As expected, at this time point, the expression of α-Syn promoted a reduction in the number of TH-positive neurons in the PPL1 cluster (Figure 3C). However, in contrast with the previous work carried out using the same *Drosophila* strain, we observed a slight reduction, also in the PPM1/2 cluster. A similar result has been described in the presence of other α-Syn-expressing lines [24], and they are probably due to differences in the imaging technique used to detect TH-positive neurons [17]. With the only exception of NAC, which exerted a protective effect against the dopaminergic neuronal loss observed in the PPL1 cluster, the other treatments did not substantially alter the α-Syn-related toxicity.

### 3.4. α-Syn Aggregation

**Figure 4:**
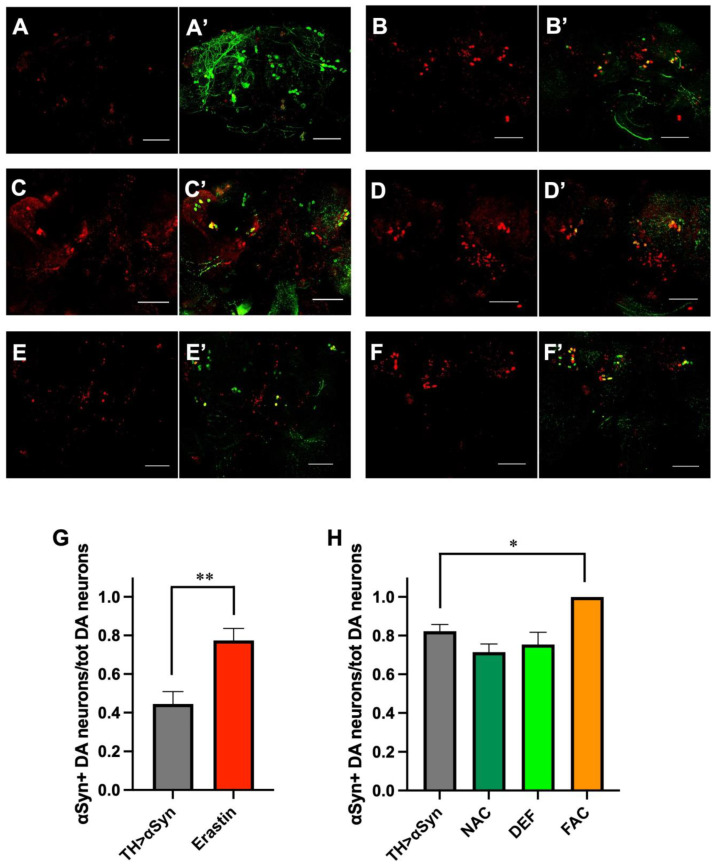
Ferroptosis modulators affect α-Syn aggregation. (**A**–**F**) Representative immunofluorescent images were acquired in 1-week-old (**A**,**A’**) and 3-week-old (**B**,**B’**) untreated α-Syn over-expressing flies, in 1-week-old α-Syn over-expressing flies treated with Erastin (**C**,**C’**), in 3-week-old α-Syn over-expressing flies treated with FAC (**D**,**D’**), NAC (**E**,**E’**) and DEF (**F**,**F’**). The red signal corresponds to α-Syn aggregates and the green signal to dopaminergic (DA) neurons. Scale bar 50 μm. (**G**) Quantitative analysis (mean ± SEM) was performed in 1-week-old untreated αSyn-overexpressing flies and following the treatment with Erastin. The statistical difference was assessed by the Mann–Whitney U test. N: 13 TH > αSyn, 7 Erastin. (**H**) Quantitative analysis (mean ± SEM) was performed in 3-week-old untreated αSyn-overexpressing flies and following the treatment with FAC, NAC, and DEF. Analysis of variance and Tukey’s multiple comparisons post-hoc test indicate the following statistically significant differences: * TH > αSyn vs. FAC, *** NAC vs. FAC, ** DEF vs. FAC. N: 15 TH > αSyn, 8 NAC, 8 Def, and 7 FAC. ***, **, *, indicate *p* < 0.001, <0.01, <0.05. For the sake of clarity, only the statistically significant differences between untreated α-Syn over-expressing flies and the other conditions are reported in the graph.

Increasing evidence supports the hypothesis that aggregated forms of α-Syn play an important role in the pathogenesis of PD. Here, we assessed whether the pathological phenotypes observed are in some way correlated to the formation of α-Syn aggregates in our experimental set-up. To this aim, we used a commercially available antibody that has been demonstrated to show high immunoreactivity for all forms of α-Syn aggregates, but showed very weak immunoreactivity toward monomers [25]. As represented in Figure 4A, the over-expression of α-Syn alone led to the formation of aggregates inside dopaminergic neurons already in young individuals and before the appearance of the other pathological phenotypes, even though, as previously mentioned, a trend toward neuronal loss and locomotor impairment was observed in 1-week-old flies over-expressing α-Syn. As expected, the number of dopaminergic neurons containing α-Syn aggregates increased in 20-day-old flies when both the climbing defects and the loss of dopaminergic neurons were more consistent (Figure 4B). In association with the previously described neuronal degeneration, the addition of erastin strongly enhanced the formation of α-Syn aggregates in surviving dopaminergic neurons. Moreover, we observed the presence of α-Syn corresponding signal outside dopaminergic neurons, consistent with the currently accepted view that α-Syn aggregated can be released from dying neurons (Figure 4C). Similarly to erastin, the addition of FAC also led to an increased presence of α-Syn aggregates in surviving dopaminergic neurons (Figure 4D). However, in this case, in addition to the extracellular signal, we also observed the α-Syn corresponding signal localized inside a cell, but not superimposed on the TH-relative signal. We do not have a clear explanation for this observation, which could be related to the fact that, in aged flies, the intensity of the TH corresponding signal is lower in comparison with younger individuals, as also observed in our control flies. While the addition of ferroptosis inducers strongly affected the propensity of α-Syn to form aggregates, the addition of either NAC (Figure 4E) or DEF (Figure 4F) did not show any effect, indicating that the accumulation of cytosolic α-Syn at high concentrations is sufficient per se to promote its aggregation. The quantitative analysis is represented in Figure 4G,H.

## 4. Discussion

In the present work, we assessed the interplay between α-Syn and iron in promoting PD-associated phenotypes using a *Drosophila melanogaster* model of the disease. To this aim, we exploited a previously generated strain that allows the selective expression of high α-Syn levels into dopaminergic neurons. In this fly line, it has been previously demonstrated that the over-expression of the protein results in progressive dopaminergic neuron cell death [17]. Here, we confirmed the data, and we also demonstrated that dopaminergic neuronal degeneration is associated with locomotor impairment and lifespan reduction. Interestingly the pathological phenotypes were emphasized by the presence of either FAC or erastin, while they were rescued by the antioxidant molecule NAC and the iron chelator DEF. Overall, our results support the involvement of ferroptosis in the α-Syn-mediated toxicity. Actually, all these compounds are well-known modulators of this cell death pathway, and they are often used in experimental models of ferroptosis. The addition of FAC in the medium leads to increased intracellular concentrations that, in turn, promote its oxidative chemistry. A previous study already evaluated the effects of iron in flies over-expressing α-Syn, both the wild-type form and its A30P and A53T pathological mutants, observing a strong iron-induced enhancement of the protein toxicity [26]. However, in that work, a higher concentration of FAC (15 mM) was used with respect to our study. Moreover, FAC was added to filter paper without other nutrients, with the exception of 1% glucose, so that water evaporation may have contributed to the observed toxicity. Here to focus as much as possible on iron-related toxicity and to limit the potential aspecific effects of iron in other tissues, we incubated flies with a lower concentration (5 mM) of FAC by adding it to the standard food. It has been previously demonstrated that the addition of 5 mM FAC induces an eight-fold increase in iron content with respect to standard food [18]. The toxic effects promoted by iron were further confirmed by treating flies with erastin, the molecule that was first associated with ferroptosis [5]. Erastin toxicity is mainly due to its ability to inhibit the system X_c_^−^, which transports cystine from the extracellular fluid into the cytosol in exchange for glutamate, causing a decreased availability of cysteine for the synthesis of GSH. This, in turn, reduces the GSH-dependent GPX4-mediated protection against lipid peroxidation. [22]. Accordingly, the treatment with NAC, a precursor for GSH biosynthesis under conditions of GSH deficiency [23], was able to rescue most of the effects induced by the over-expression of α-Syn. Our results agree with previous data showing that alterations in GSH metabolism can modulate α-Syn-dependent injury in *Drosophila melanogaster* [17]. More specifically, while a 45% reduction in GSH levels was associated with an increased loss of TH-positive neurons, higher concentrations of glutathione completely rescued the α-Syn-dependent neuronal loss [17]. Furthermore, the over-expression of α-Syn in a *glutathione-S-transferase* (*GstS1*) null background induced an enhanced dopaminergic neuronal degeneration with respect to flies over-expressing α-Syn alone, while the toxic phenotype was suppressed when GstS1 was over-expressed in the dopaminergic neurons together with α-Syn [17].

Using a commercially available antibody, designed to specifically detect both α-Syn oligomers and fibrils [25], in the present study, we also evaluated the correlation between the α-Syn-induced toxic phenotypes and the appearance of α-Syn aggregates, as well as the modulatory effects of the previously described molecules. The over-expression of α-Syn in our fly model induced the accumulation of protein aggregates inside dopaminergic neurons in a time-dependent way, their amount being higher 20 days after eclosion than in 5-day-old flies. Interestingly, while both FAC and erastin strongly promoted α-Syn aggregation, any significant effect was observed in the presence of the anti-ferroptosis compounds. These results suggest that high levels of cytosolic α-Syn are sufficient *per se* for the formation of aggregates, while both iron accumulation and the oxidative conditions derived from GSH depletion accelerate their formation, exacerbating their toxicity. Moreover, our data also indicate that, most likely, both NAC and DEF protect against downstream effects mediated by α-Syn aggregates, such as lipid peroxidation or intracellular reactive oxygen species (ROS) formation. Our results are consistent with recent analyses carried out in vitro in cellular models of α-synucleinopathies where the toxicity of the α-Syn aggregates has been thoroughly explored. First, in human iPSC-derived neurons with *SNCA* gene triplication, the increased expression of α-Syn led to the generation of ROS, increased lipid peroxidation, and made neurons more vulnerable to exogenously applied α-Syn oligomers, supporting the involvement of α-Syn aggregates in mediating α-Syn toxicity [27,28]. Interestingly, the oligomer-induced ROS production was demonstrated to be entirely dependent on the presence of free metal ions [27]. Moreover, while the treatment with erastin enhanced cell death in a dose-dependent way, the oligomer-induced neuronal loss was reduced to basal levels by the addition in the growth medium of the iron chelator desferoxamine, the inhibitor of lipid peroxidation D4-Lnn (deuterated linoleic acid) or the lipophilic antioxidant ferrostatin [28], suggesting that ferroptosis is involved in the toxic effects mediated by α-Syn aggregates and, in particular, α-Syn oligomers. Similar indications were more recently obtained in human midbrain dopaminergic neurons, where SNCA gene triplication caused an increased vulnerability to lipid peroxidation and ferroptosis induced through the RSL3-mediated inhibition of GPX4 [29]. Interestingly, the same investigators also demonstrated that a reduction in α-Syn expression was able to protect neurons from ferroptosis-induced lipid peroxidation and subsequent cell death [29], indicating that the presence of the protein is necessary for, or it can strongly exacerbate, the iron-dependent cell death pathway. Consistent with these results, the downregulation of α-Syn in SH-SY5Y cells was shown to reduce iron toxicity. Moreover, in the same cellular model, α-Syn mRNA and protein levels were found to increase significantly in iron-treated cells compared to control cells [30]. Increased accumulation/aggregation of α-Syn in the presence of FAC or erastin has been suggested to result from ROS-mediated inactivation of the proteasomal degradation system that is primarily responsible for the clearance of alpha-syn from inside the cells [30]. Additionally, iron could enhance the translation of α-Syn mRNA through an iron-responsive element, which has been described in the 5′-untraslated region of α-Syn mRNA [31]. Overall, these results suggest that high levels of iron could promote first α-Syn accumulation and then its aggregation.

Collectively, the results presented in this work, together with the data that are emerging in the literature, support the involvement of ferroptosis in the pathogenesis of PD, and this regulated cell death pathway could account for the two pathological hallmarks of the disorder. The first one is the preferential degeneration of dopaminergic neurons, and there is now a general consensus concerning the role of dopamine itself in promoting oxidative conditions, making this neuronal population more prone to oxidative damage [32,33]. The second main feature is the presence of Lewy bodies, mainly composed of fibrillar α-Syn, which support a role for the protein in the disease onset and progression. Interestingly, high expression levels of α-Syn seem to correlate directly with its neurotoxicity. In fact, some genetic forms of PD are associated with *SNCA* gene duplication and triplication [34,35,36,37], and variations in the *SNCA* gene that lead to increased protein expression levels represent a genetic risk factor for sporadic PD [38]. In this frame, age-related alterations of iron levels in dopaminergic neurons of Substantia nigra pars compacta, which are further exacerbated in PD brains [9,11,39], might promote both dopamine oxidative reactions and α-Syn aggregation. It is worth mentioning that, while this study specifically focused on the possible involvement of ferroptosis in α-Syn-dependent toxicity, other cell death pathways may be involved as well. More specifically, as reviewed elsewhere, intrinsic and extrinsic caspase-dependent apoptosis and autophagic cell death appear as the most supported pathways in regard to PD [40]. In this context, it may also be pointed out that, in human iPSC-derived neurons with *SNCA* triplication, the iron-dependent production of ROS was shown to cause cell death with some features of apoptosis [27]. In the future, it will be interesting to understand whether these multiple cell death pathways are converging to induce Substantia nigra neurodegeneration, or if they all act independently. In conclusion, although additional studies are required to further confirm our results in other animal models, the involvement of ferroptosis in PD could open the field to novel therapeutic approaches.

## Data Availability

Data is contained within the article.
